# Apraxic deficits predict general cognitive impairment in patients with biomarker-verified Alzheimer’s pathology

**DOI:** 10.1007/s00415-025-13401-9

**Published:** 2025-09-25

**Authors:** Claudia C. Schmidt, Michella M. Bardakan, Elena Jaeger, Nils Richter, Gérard N. Bischof, Kathrin Giehl, Oezguer A. Onur, Frank Jessen, Gereon R. Fink, Alexander Drzezga, Peter H. Weiss

**Affiliations:** 1 Cognitive Neuroscience, Institute of Neurosciences and Medicine (INM-3), Forschungszentrum Jülich, Jülich, Germany; 2 Department of Nuclear Medicine, Faculty of Medicine, University Hospital Cologne, University of Cologne, Cologne, Germany; 3 Department of Neurology, Faculty of Medicine, University Hospital Cologne, University of Cologne, Cologne, Germany; 4 Molecular Organisation of the Brain, Institute of Neurosciences and Medicine (INM-2), Forschungszentrum Jülich, Jülich, Germany; 5 Department of Psychiatry and Psychotherapy, Faculty of Medicine, University Hospital Cologne, University of Cologne, Cologne, Germany; 6 German Centre for Neurodegenerative Diseases (DZNE), Bonn-Cologne, Bonn, Germany

**Keywords:** Limb apraxia, Gesture imitation, Pantomime, Mini-Mental State Examination (MMSE), Alzheimer’s disease

## Abstract

Apraxia represents a core feature of Alzheimer’s disease (AD), a neurodegenerative disorder characterised by the accumulation of β-amyloid plaques and tau deposition. However, systematic descriptions of apraxic deficits in AD patients remain scarce. Here, we comprehensively investigate apraxia profiles and their link with cognitive impairment in patients with biomarker-verified Alzheimer’s pathology. We characterised the frequency and patterns of apraxic deficits in patients with biomarker-verified Alzheimer’s pathology using a battery of standardised apraxia tests. Demographic variables and apraxia scores were related to patients’ general cognitive impairment using hierarchical regression analysis. Apraxic deficits were found in 67% of patients with biomarker-verified Alzheimer’s pathology (*n* = 63). Patients with Alzheimer’s pathology were more impaired in imitating finger gestures (than hand gestures: 89.2% vs. 80.0%, *p* < 0.001) and imitating complex hand movements (than single hand movements: 97.4% vs. 78.5%, *p* < 0.001), even when controlling for general cognitive impairment. Apraxia assessments explained about 60% of the variance in dementia severity, with performance in the KAS subtest of pantomiming object use (beta coefficient: 0.47, *p* = 0.001) and the DATE subtest for limb apraxia (beta coefficient: 0.37, *p* = 0.005) constituting significant predictors of general cognitive impairment. These findings emphasise the relevance of apraxia in patients with biomarker-verified Alzheimer’s pathology, revealing that praxis deficits predict general cognitive impairment in AD. Further research is warranted into the role of apraxia as a potential early diagnostic criterion in AD.

**Supplementary Information:**

The online version contains supplementary material available at 10.1007/s00415-025-13401-9.

## Introduction

Alzheimer’s disease (AD) is a neurodegenerative disorder characterised by a progressive decline in cognitive functions, particularly episodic memory [1]. Importantly, current diagnostic criteria for AD mark the presence of atypical, non-amnestic neuropsychological symptoms [2]. Among these, apraxia represents a critical cognitive phenotype that warrants further investigation [3] since it impacts the activities of daily living: Apraxia impairs cognitive–motor functions, including gesture imitation, pantomiming object use, and actual object use, and cannot be (solely) attributed to basic motor deficits [4]. Apraxic deficits constitute a core feature of AD that increases in severity [5] as AD progresses. Likewise, the prevalence of apraxic deficits increases from around 30% in mild cases of AD to 90% in severe stages of the disease [3, 6].

Notably, the presence of (limb) apraxia has been shown to distinguish between AD and frontotemporal dementia [7], subcortical vascular dementia, and mild cognitive impairment (MCI) [8]. Already in the early stages of AD, apraxia supports the differential diagnosis of Alzheimer’s dementia from other dementia subtypes, as AD patients present with distinctive clinical patterns of apraxic deficits [9–11]. In particular, patients with AD exhibited pronounced deficits in imitating hand and finger gestures, whereas patients with frontotemporal dementia showed specific impairments in imitating bucco-facial gestures [9, 10]. In patients with frontotemporal dementia, the AD apraxia profile (i.e. more severe deficits in limb apraxia compared to bucco-facial apraxia) was associated with cerebrospinal fluid (CSF)-based biomarkers indicative of additional AD pathology [12].

Despite the documented importance of apraxic deficits in AD, systematic investigations on the frequency and patterns of apraxic deficits in AD remain scarce, particularly in patients with biomarker-verified Alzheimer’s pathology. According to the current diagnostic criteria of the National Institute on Aging and the Alzheimer’s Association (NIA-AA) [2], the diagnosis of AD includes specific neuropathological markers, namely amyloid-β (A +) and (phosphorylated) tau (T +), which can be detected in the CSF or in the brain by positron emission tomography (PET) [13]. This biomarker-based definition of AD is considered crucial for reliably distinguishing between AD and other neurodegenerative diseases that can lead to dementia [14].

In the present study, we comprehensively characterised apraxia in a well-defined sample of patients with Alzheimer’s pathology confirmed by CSF- or PET-derived biomarkers (i.e. amyloid-β (A +) and tau (T +)). To this end, we investigated the frequency and severity of apraxic deficits and their relationship to the patient’s cognitive status. Moreover, we probed the presence of differential patterns of praxis deficits (i.e. apraxia profiles) in the patients with Alzheimer’s pathology, while controlling for general cognitive deficits. Building on previous studies showing more pronounced deficits in imitating limb gestures than facial gestures in AD patients [9], we further explored putative differential impairments in imitating hand versus finger gestures in patients with biomarker-verified Alzheimer’s pathology. Neuropsychological studies in stroke patients have shown that patients with unilateral right hemisphere damage were impaired specifically in the imitation of finger gestures (compared to healthy control subjects). In contrast, patients with unilateral left hemisphere strokes had similar deficits in imitating finger gestures as patients with right hemisphere strokes, but suffered from additional deficits in imitating hand gestures [15]. Since AD pathology typically occurs bilaterally, we hypothesised that the patients with Alzheimer’s pathology would be more impaired in imitating finger gestures than hand gestures. We also hypothesised that patients with Alzheimer’s pathology would perform worse in imitating complex movements compared to single gestures. Besides, we tested whether the effect of movement complexity is modulated by the effector performing the movements (i.e. hand vs. finger). Finally, by investigating whether apraxic deficits predict general cognitive impairment, this study provided insights into the potential predictive value of praxis performance (or deficits thereof) for cognitive functioning in patients with biomarker-verified Alzheimer’s pathology.

## Methods

### Patient sample

One hundred twenty-three patients with clinically suspected AD were recruited from the Centre for Memory Disorders (ZfG; ‘Zentrum für Gedächtnisstörungen’) of the departments of Neurology and Psychiatry at the University Hospital Cologne. Patients were included in the study if they were at least 50 years old, met the NIA-AA criteria for the diagnosis of Alzheimer’s clinical syndrome [2, 16], and had biomarker-confirmed amyloid and tau pathology based on CSF and/or PET imaging according to current diagnostic guidelines [14]. Patients were excluded if they (i) were clinically diagnosed with dementia other than of the Alzheimer’s disease type, (ii) suffered from other conditions potentially responsible for cognitive decline or motor deficits (e.g. cerebrovascular disorders, Parkinson’s disease, multiple sclerosis), (iii) showed visual or auditory deficits interfering with neuropsychological testing, or (iv) were unable to give informed consent.

All patients provided written informed consent before the study. The local ethics committee approved the study.

### Determination of CSF and PET biomarker status

Patients’ CSF was analysed for AD biomarkers, i.e. pathologically decreased CSF β-amyloid 42 [Aβ_42_] levels or decreased Aβ_42_/Aβ_40_ ratio [A +] and pathologically increased CSF phosphorylated tau levels [T +] [14]. CSF concentrations of β-amyloid 42 levels [Aβ_42_], β-amyloid 40 levels [Aβ_40_], and phosphorylated tau [p-tau] were quantified using the ELISA assay (Euroimmun) or Elecsys assay (Roche). The following thresholds were used to determine positivity on Aβ_42_ or Aβ_42_/Aβ_40_ ratio, and p-tau levels: *Euroimmun*: Aβ_42_ < 629 pg/mL, Aβ_42_/Aβ_40_ ratio < 0.095, p-tau protein > 61 pg/mL; *Roche*: Aβ_42_ < 1,030 pg/mL, p-tau ≥ 27 pg/mL.

Patients’ PET scans were rated for cerebral AD pathology, i.e. as amyloid- or tau-positive. Amyloid or tau PET imaging was performed using the following tracers: *amyloid PET*: [^18^F]Florbetaben or [^11^C]PiB; *tau PET*: [^18^F]PI-2620 or [^18^F]AV-1451.

### Clinical and apraxia assessment

All patients underwent a detailed clinical work-up at the outpatient memory clinic of the University Hospital Cologne (i.e. Centre for Memory Disorders (ZfG; ‘Zentrum für Gedächtnisstörungen’)). The diagnosis of AD with MCI or dementia was based on a comprehensive neurological examination, including medical history, cerebral imaging, and blood tests. The latter were also used to rule out reversible causes of dementia. In addition, all patients underwent a thorough neuropsychological assessment by a neurologist, psychiatrist, or psychologist experienced in the diagnosis and treatment of neurodegenerative diseases.

The patients’ overall cognitive status was assessed using the German version of the Mini-Mental State Examination (MMSE) [17] that evaluates impairments in general cognitive functions, including orientation, attention, working memory, language, and delayed recall [18].

As apraxia represents a multi-componential syndrome comprising different impaired motor-cognitive processes [19], patients’ praxis functions were assessed using the following established apraxia tests: the Cologne Apraxia Screening (Kölner Apraxie Screening, KAS) [20], the Dementia Apraxia Test (DATE) [10], the imitation tests by Goldenberg [15], a modified version of the De Renzi test for imitation [21], and a modified version of the De Renzi test for actual object use [22].

The KAS is a standardised, validated diagnostic instrument initially designed to screen for apraxia in stroke patients [20]. It has also been effectively used to diagnose apraxia in mild dementia [23]. It comprises two subtests to assess pantomime of object use and two subtests to assess gesture imitation. In the pantomime subtests, patients are shown photographs of everyday objects and asked to demonstrate their typical use involving bucco-facial movements (5 items; e.g. pantomiming the use of a toothbrush) and arm/hand movements only (5 items; e.g. pantomiming the use of scissors). In the imitation subtests, patients are presented with photographs showing a woman performing five bucco-facial gestures (e.g. sticking out her tongue) and five arm/hand gestures (e.g. making the stop sign with her hand) and asked to reproduce these gestures. The maximum total KAS score is 80 points (40 points for the pantomime test and 40 points for the imitation test); a score of 76 points or less indicates apraxia [20].

The DATE is a validated clinical screening instrument for praxis impairments in neurodegenerative diseases [10]. It consists of five subtests, two assessing limb apraxia and three assessing bucco-facial apraxia. For limb apraxia, the imitation of hand and finger postures (8 items) and pantomiming of object use (2 items) are tested. For bucco-facial apraxia, the subtests assess imitation of facial postures (6 items), production of emblematic bucco-facial postures upon verbal command (2 items; e.g. “show me how you clear your throat”), and repetition of pseudowords to test for apraxic speech (2 items). The maximum total DATE score is 60 points (30 points for limb apraxia and 30 points for bucco-facial apraxia); a score below 46 points suggests apraxia [10].

The Goldenberg imitation tests assess the imitation of hand positions (10 items) and finger configurations (10 items) demonstrated by the examiner. Each imitation test has a maximum score of 20 points, with a score below 18 points in the hand imitation test and below 17 points in the finger imitation test marking apraxic imitation deficits [15].

The De Renzi test for imitation comprises four subtests assessing the imitation of single (static) hand postures (6 items) and finger configurations (6 items), as well as complex sequences of hand (6 items) and finger movements (6 items). The maximum test score is 72 points (18 points for each subtest), with a score below 53 points indicating apraxia [21].

The De Renzi test for actual object use assesses the use of single objects (5 items) and tool-object pairs (2 items), with scores below 30 points (out of a maximum of 32 points) revealing apraxia [22].

Raw scores of the apraxia tests were converted into percentages of the maximum score to account for the different score ranges.

### Statistical analysis

We performed statistical analyses using IBM SPSS Statistics (Statistical Package for the Social Sciences, version 28). For each patient, we calculated an overall apraxia severity index based on the number of impaired apraxia tests, which categorised patients into groups with no apraxia (0 tests impaired), mild apraxia (1–2 tests impaired), moderate apraxia (3–4 tests impaired), or severe apraxia (5–6 tests impaired). Nonparametric Spearman correlation analyses examined the relationship between the severity of apraxia and demographic factors (age, years of education) and cognitive impairment (indexed by the MMSE score).

To test for a difference in imitating hand and finger gestures independent of the patients’ general cognitive status, we analysed the mean imitation performance (in %) in the Goldenberg imitation tests in a mixed model analysis of covariance (ANCOVA) with *effector* (hand and finger) as within-subject factor and the MMSE scores as a covariate to control for general cognitive impairment. Besides, we explored putative differences in imitating single versus complex hand and finger movements (in %) in the De Renzi imitation test using an ANCOVA with *effector* (hand and finger) and *complexity* (single and complex) as within-subject factors. We included the patients’ MMSE score as a covariate to control for general cognitive impairment. A significant interaction was assessed post hoc by simple main effects controlling for general cognitive impairment.

To investigate whether demographic factors (i.e. age or years of education) or praxis performance can explain variance in cognitive impairment in patients with Alzheimer’s pathology, we computed a hierarchical linear regression analysis. This analysis defined two models with the MMSE score as the dependent variable. The first-level model included age and years of education as predictive variables. In the second-level model, we added simultaneously the scores (in %) in the two KAS subtests of pantomiming object use and imitating gestures, the two DATE subtests for limb and bucco-facial apraxia, the difference score between the imitation of hand positions and finger configurations in the Goldenberg imitation tests, and an interaction score for the differential performance in imitating single versus complex hand and finger gestures in the De Renzi imitation test to the regression model. We did not include the De Renzi test for actual object use in the regression analysis as it was the least sensitive apraxia test in our cohort of patients with Alzheimer’s pathology. We estimated the two models’ fit by calculating the Akaika Information Criterion (AIC), a goodness of fit measure that corrects for model complexity by penalising increasing numbers of predictors [24]. We used the AICs to compare both levels of the regression model and to verify that a larger number of predictors did not merely drive the increase in predictive power of the second-level model.

We set a significance level of *p* < 0.05 (two-sided) for all analyses.

## Results

### Patient sample characteristics

The current study included 63 patients (29 women) with biomarker-confirmed Alzheimer’s pathology based on at least one of the two diagnostic modalities, CSF or PET imaging, i.e. abnormal amyloid-β (A +) and tau protein levels (T +) [14]. Alzheimer’s pathology (A +/T +) was confirmed in 28 patients by CSF, in 20 patients by CSF and tau PET, and in 10 patients by (amyloid and tau) PET. In the remaining patients, Alzheimer’s pathology was confirmed by variable combinations of CSF and PET imaging. Please refer to the **Suppl. Table** for an overview of amyloid and tau abnormalities for each patient included in the study. The group of patients had a mean age of 70.1 years (standard deviation [SD] = 9.9, range 50–87) and a mean education level of 14.7 years (SD = 3.4, range 8–21).

Based on the NIA-AA criteria [2, 16], 40 patients (63%) were clinically diagnosed with mild cognitive impairment (MCI), 22 patients (35%) with mild dementia, and one patient with moderate dementia. All patients showed impairments in episodic memory, i.e. suffered from an amnestic variant of AD. All patients with dementia and 28 of the patients with MCI were impaired in at least one other cognitive domain besides episodic memory. In the remaining 12 MCI patients, impairment of episodic memory predominated (see **Suppl. Table** for an overview of the clinical diagnosis in the patients with Alzheimer’s pathology).

The mean MMSE score of the patient group was 22.5 points (SD = 5.4, range 10−30). Note that 29% of the patients (*n* = 18) had a normal MMSE score (≥ 27/30 points) despite positive AD biomarkers.

### Frequency and severity of apraxic deficits

In the current sample of patients with biomarker-verified Alzheimer’s pathology, 67% (*n* = 42/63) were apraxic in at least one apraxia test (see Table 1 for an overview of the patients’ performance on the different apraxia tests). Of those, 48% (*n* = 20) had mild apraxia, 21% (*n* = 9) had moderate apraxia, and 31% (*n* = 13) suffered from severe apraxia. The KAS and DATE were the most sensitive tests for detecting apraxia in 59% (*n* = 37) and 37% (*n* = 23) of the patients with Alzheimer’s pathology, respectively. In contrast, the De Renzi test for actual object use showed the lowest sensitivity, detecting apraxic deficits in only 6% (*n* = 4) of the patients.

**Table 1:** Praxis assessments in patients with biomarker-verified Alzheimer’s pathology (*n* = 63)

Apraxia test	Praxis performance (in %)	Cut-off scores	Number of patients impaired
KAS (total score)	88.6 ± 14.7 (25–100)	≤ 95%	37
DATE (total score)^#^	76.8 ± 19.1 (15.8–100)	≤ 75%	23
Goldenberg finger imitation test	80.0 ± 22.4 (0–100)	< 85%	23
Goldenberg hand imitation test	89.2 ± 14.7 (20–100)	< 90%	20
De Renzi test for imitation^#^	81.6 ± 16.9 (8.3–100)	< 72.2%	16
De Renzi test for actual object use^#^	97.4 ± 9.3 (31.3–100)	≤ 94%	4

The mean ± standard deviation and range (in parentheses) are given. All apraxia scores were converted into percentages of the maximum score for comparability

^#^ *n* = 62

*DATE* Dementia Apraxia Test; *KAS* Kölner (Cologne) Apraxia Screening

As the KAS was initially developed to assess apraxic deficits in left hemisphere stroke patients [20], we evaluated its relationship to the DATE, a validated test for the diagnosis of apraxia in various forms of dementia [10]. The KAS total score showed a strong positive correlation with the DATE total score (*ρ* = 0.718, *p* < 0.001). Likewise, the KAS and DATE were significantly associated (*χ*^*2*^ = 12.54, *p* < 0.001): approximately 87% of the apraxic patients according to the DATE also demonstrated apraxic deficits per the KAS. Further, 56% of the patients showing apraxic deficits according to the KAS were also classified as apraxic per the DATE. In line with the higher sensitivity of the KAS for revealing apraxic deficits in patients with Alzheimer’s pathology, 26% (*n* = 16) of the patients who showed apraxic deficits as revealed by the KAS did not show apraxic deficits in the DATE. In contrast, only 5% (*n* = 3) of all cases showed apraxic deficits according to the DATE but not the KAS.

### Relationship between apraxia and general cognitive impairment

The severity of apraxia (indexed by the number of impaired apraxia tests) correlated significantly with the severity of cognitive impairment (assessed by the MMSE) in the patients with Alzheimer’s pathology (*ρ* = -0.59, *p* < 0.001): the more severe the cognitive impairment (i.e. the lower the MMSE score), the more apraxia tests were impaired.

Notably, there were no significant correlations between apraxia severity and age (*ρ* = 0.07, *p* = 0.590) or years of education (*ρ* = 0.04, *p* = 0.751) in the current sample of patients with Alzheimer’s pathology.

### Apraxia profiles (controlled for general cognitive impairment)

**Fig. 1:**
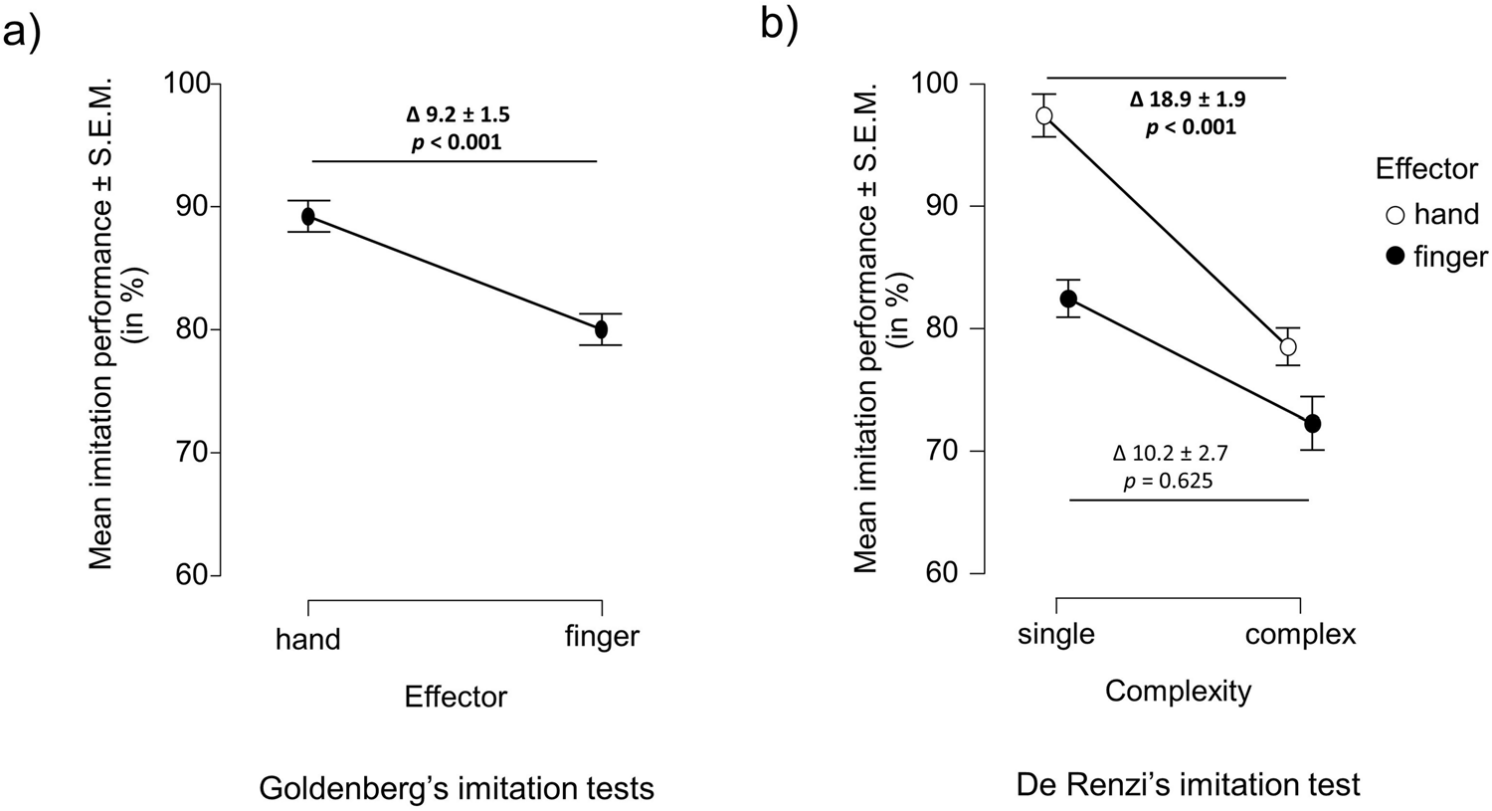
Differential patterns of apraxic imitation deficits in patients with biomarker-verified Alzheimer’s pathology. **a** Patients with Alzheimer’s pathology (*n* = 63) showed significantly worse performance in imitating finger configurations compared to hand postures in the imitation tests by Goldenberg, even after controlling for general cognitive impairment (assessed by the MMSE). **b** Patients with Alzheimer’s pathology (*n* = 62) imitated complex hand movements worse than single hand movements, while there was no significant difference in imitating single and complex finger gestures in the De Renzi imitation test after controlling for general cognitive impairment (assessed by the MMSE). *MMSE* Mini-Mental State Examination. Error bars indicate the standard error of the mean (S.E.M.)

The ANCOVA testing for a difference in performance in imitating hand and finger gestures revealed a significant main effect of *effector*, even after controlling for general cognitive impairment (*F*_(1,61)_ = 40.49, *p* < 0.001): patients with Alzheimer’s pathology imitated finger configurations worse than hand positions (89.2% vs. 80.0%; Fig. 1a) in Goldenberg’s imitation tests.

The ANCOVA that tested for a difference in the imitation of single versus complex hand and finger gestures in De Renzi’s imitation test (while controlling for general cognitive impairment) showed a significant main effect of *complexity* (*F*_(1,60)_ = 13.27, *p* < 0.001) besides a significant main effect of *effector* (*F*_(1,60)_ = 5.31, *p* = 0.025). Notably, there was also a significant *effector* x *complexity* interaction effect (*F*_(1,60)_ = 16.61, *p* < 0.001), which revealed that complex hand gestures were imitated worse than single hand gestures (*F*_(1,60)_ = 42.66, *p* < 0.001; 97.4% vs. 78.5%), while there was no significant difference in imitating single and complex finger gestures (*F*_(1,60)_ = 0.24, *p* = 0.625; 82.4% vs. 72.2%; Fig. 1b) after controlling for general cognitive impairment.

### Predicting general cognitive impairment by apraxic deficits

The hierarchical regression analysis revealed that a model including age and years of education did not explain the variance in general cognitive impairment among patients with Alzheimer’s pathology (adjusted *R*^2^ = 0.02, *F*_(2,59)_ = 0.51, *p* = 0.606). The predictive value of the model improved significantly when scores of the apraxia assessments were added to age and educational years (adjusted *R*^2^ = 0.59, *F*_(8,53)_ = 12.13, *p* < 0.001; *R*^2^ change: *F*_(6,53)_ = 15.75, *p* < 0.001). Of the eight predictors, performance in the KAS subtest of pantomiming object use (beta = 0.47, *t* = 3.40, *p* = 0.001) and the DATE subtest for limb apraxia (beta = 0.37, *t* = 2.96, *p* = 0.005) were independent predictors of general cognitive impairment severity. Estimation of the models’ fit revealed that the first-level (two predictors) model had an AIC of 208.76, and the second-level (eight predictors) model had an AIC of 157.31. Since the model with a lower AIC had a better fit, the second-level model including apraxia scores was the preferred model. This finding also indicated that the increased predictive power of the second-level model was not simply due to the benefit of the additional variables per se but was instead specific to apraxic deficits.

## Discussion

This study investigated the frequency and patterns of apraxic deficits and their association with cognitive impairment in 63 patients with biomarker-verified Alzheimer’s pathology, i.e. patients with abnormal amyloid-β and tau protein levels (A +/T +) in the CSF or PET imaging. Considering diverse praxis functions, our study revealed a high prevalence of apraxia: 67% of the patients showed apraxic deficits in at least one apraxia test, and about half of the patients even suffered from moderate to severe apraxia. This finding suggests that apraxia is common already in the mild (to moderate) stages of AD, as most of the current patients presented with MCI or mild dementia. In line with previous reports [25], apraxia severity was related to the severity of general cognitive impairment.

As hypothesised, patients with Alzheimer’s pathology showed greater impairment in imitating finger configurations compared to hand positions, independent of general cognitive deficits. This finding is consistent with previous neuropsychological studies in stroke patients with apraxia, reporting dissociations between the imitation of hand and finger gestures [26, 27], suggesting different underlying cognitive processes. It has been proposed that imitating hand gestures primarily relies on the processing of spatial relationships between different body parts. In contrast, imitating finger gestures puts higher demands on visuo-spatial control for perceptual discrimination between relatively uniform elements of the body (i.e. fingers) [19, 28]. In line with this, deficits in imitating finger gestures were associated with visuo-constructive deficits in a previous study of patients with dementia [29]. Moreover, poorer performance when imitating finger configurations (compared to hand positions) has often been found in patients with right hemisphere stroke [30], probably due to deficits in visuo-spatial processing [31–33].

Our study also revealed that the effector performing the gesture (i.e. hand vs. finger) modulated the effect of movement complexity (i.e. worse performance in imitating complex movements compared to single gestures): patients with Alzheimer’s pathology were more impaired in imitating complex compared to single *hand* movements. In contrast, the difference in imitation performance between complex and single *finger* movements was less pronounced and no longer statistically significant when controlling for general cognitive impairment. Thus, complex hand movements (as opposed to single hand movements) showed enhanced sensitivity in detecting imitation deficits in patients with Alzheimer’s pathology. The results complement previous studies, which showed marked difficulties in imitating spatially complex (bimanual) or sequential movements in patients with AD [29, 34].

Finally, our study showed that praxis performance, but not age or years of education, predicted general cognitive impairment in patients with biomarker-verified Alzheimer’s pathology. A regression model with age and educational years explained only 2% of the variance in general cognitive deficits. Adding apraxia scores improved the model to about 60% explained variance, which represented a statistically significant increase in predictive power. The finding that performance in the KAS subtest of pantomiming object use and the DATE subtest for limb apraxia (involving imitation of hand and finger gestures and pantomime of object use) were independent predictors of general cognitive functioning supports and extends the relevance of limb apraxia as a marker of cognitive impairment in patients with Alzheimer’s pathology, as implicated by several previous studies in patients with mild-to-moderate AD [10, 11, 35]. It further demonstrates that deficits in pantomiming object use are a significant predictor of general cognitive impairment. It has been proposed that several cognitive mechanisms underlie the pantomime of object use, including semantic knowledge about tool function, sensorimotor knowledge about tool manipulation, and mechanical knowledge for technical reasoning about object use [36]. Both semantic knowledge and manipulation knowledge appear to be impaired even in mild stages of AD [3], likely reflecting their dependence on temporal and parietal lobe structures, which are affected by neurodegeneration early in the course of AD [37, 38]. Consistent with this, grey matter atrophy in the middle temporal gyrus and angular gyrus has been found to be associated with deficits in pantomiming object use in the early stages of AD [23]. Moreover, tau aggregation in temporal, parietal, and occipital regions has recently been associated with apraxic deficits in patients with biomarker-confirmed diagnosis of AD [39]. Accordingly, our data suggest that impaired praxis performance may serve not only as a predictor of general cognitive impairment but also as an (early) behavioural marker of Alzheimer’s pathology.

In conclusion, the present study established that in patients with biomarker-verified Alzheimer’s pathology apraxic deficits are common even at mild (to moderate) disease stages and show distinct patterns in different praxis domains. The apraxic deficits explained variance in the patients’ general cognitive impairment, emphasising the relevance of apraxia in AD. Our results warrant further investigation into the role of apraxia as a potential behavioural marker of Alzheimer’s pathology at early stages of AD.

## Supplementary Information

Below is the link to the electronic supplementary material.

Supplementary file1 (DOCX 29 KB)

## Data Availability

The data supporting this study’s findings are not publicly available due to the ethical consensus on data protection approved by the local ethics committee and signed by the patients. The authors may provide the data upon reasonable request.
