# Supplementary material for: Apraxic deficits predict general cognitive impairment in patients with biomarker-verified Alzheimer’s pathology
Source: J Neurol. 2025 Sep 25;272(10):654. doi: 10.1007/s00415-025-13401-9 (PMC12464036; doi:10.1007/s00415-025-13401-9)
Supplement: Supplementary file 1 — Supplementary file1 (DOCX 29 KB) [file 415_2025_13401_MOESM1_ESM.docx]

**Supplementary Material**

**Suppl. Table: Overview of amyloid and tau abnormalities and clinical diagnosis in patients with Alzheimer’s pathology (n = 63)**

| **Patient** | **Amyloid+ (CSF) ↓** | **Tau+ (CSF) ↑** | **Amyloid+ (PET)** | **Tau+ (PET)** | **Clinical diagnosis** |
| --- | --- | --- | --- | --- | --- |
| 1 | + | + | NA | + | Mild dementia |
| 2 | + | + | NA | NA | Mild dementia |
| 3 | + | + | NA | NA | MCI; single-domain |
| 4 | + | + | NA | + | MCI; single domain |
| 5 | + | + | NA | + | MCI; multi-domain |
| 6 | + | + | NA | + | MCI; multi-domain |
| 7 | + | + | NA | NA | MCI; multi-domain |
| 8 | + | + | NA | NA | Mild dementia |
| 9 | + | + | NA | − | MCI; multi-domain |
| 10 | + | + | NA | + | Mild dementia |
| 11 | NA | NA | + | + | Mild dementia |
| 12 | + | + | NA | NA | MCI, single-domain |
| 13 | + | + | NA | + | MCI; multi-domain |
| 14 | NA | NA | + | + | Mild dementia |
| 15 | NA | NA | + | + | MCI; multi-domain |
| 16 | NA | NA | + | + | Mild dementia |
| 17 | + | + | NA | + | MCI; multi-domain |
| 18 | NA | NA | + | + | MCI; multi-domain |
| 19 | + | + | NA | + | MCI; multi-domain |
| 20 | NA | NA | + | + | Mild dementia |
| 21 | + | + | + | NA | MCI; single-domain |
| 22 | NA | NA | + | + | MCI; multi-domain |
| 23 | + | + | NA | + | Mild dementia |
| 24 | + | + | + | + | MCI; multi-domain |
| 25 | + | + | NA | + | MCI; multi-domain |
| 26 | + | + | NA | NA | MCI; multi-domain |
| 27 | + | + | NA | + | Mild dementia |
| 28 | + | + | NA | NA | MCI; multi-domain |
| 29 | + | + | NA | NA | Mild dementia |
| 30 | + | + | NA | NA | Mild dementia |
| 31 | + | + | NA | + | Mild dementia |
| 32 | + | + | + | + | MCI; multi-domain |
| 33 | + | + | NA | NA | MCI; single-domain |
| 34 | + | + | NA | NA | Mild dementia |
| 35 | + | + | NA | NA | Mild dementia |
| 36 | + | + | NA | NA | MCI; multi-domain |
| 37 | NA | NA | + | + | MCI; multi-domain |
| 38 | + | + | NA | NA | MCI; single-domain |
| 39 | + | + | NA | + | MCI; multi-domain |
| 40 | + | + | NA | NA | MCI; multi-domain |
| 41 | + | + | NA | NA | Mild dementia |
| 42 | + | + | NA | + | MCI; single-domain |
| 43 | NA | NA | + | + | MCI; multi-domain |
| 44 | + | + | NA | NA | MCI; multi-domain |
| 45 | + | + | NA | NA | Mild dementia |
| 46 | + | + | NA | NA | Mild dementia |
| 47 | + | + | NA | + | MCI; multi-domain |
| 48 | + | + | NA | + | MCI; single-domain |
| 49 | + | + | NA | + | Mild dementia |
| 50 | NA | NA | + | + | Moderate dementia |
| 51 | + | + | NA | NA | MCI; single-domain |
| 52 | + | + | NA | NA | MCI; single-domain |
| 53 | + | + | NA | NA | MCI; multi-domain |
| 54 | + | + | NA | NA | MCI; multi-domain |
| 55 | + | + | NA | + | Mild dementia |
| 56 | + | + | NA | NA | Mild dementia |
| 57 | + | + | NA | NA | MCI; multi-domain |
| 58 | + | + | + | + | MCI; multi-domain |
| 59 | + | + | NA | + | Mild dementia |
| 60 | + | − | NA | + | MCI; single domain |
| 61 | + | + | NA | NA | MCI; single domain |
| 62 | + | + | NA | NA | MCI; multi-domain |
| 63 | + | + | NA | + | MCI; multi-domain |

The table illustrates the status of amyloid-β and tau protein biomarkers for each patient included in the study. Results are reported separately for abnormalities of amyloid and tau pathology as assessed in cerebrospinal fluid (CSF) or via positron emission tomography (PET). A ‘+’ indicates an abnormal value and a ‘-’ indicates a normal value in the conducted examination. ‘NA’ means that the corresponding diagnostics were not performed. All patients tested positive for amyloid-β (A+) and tau protein (T+) with respect to at least one of the two diagnostic modalities.

All patients showed impairments in (episodic) memory, i.e., suffered from an amnestic variant of Alzheimer’s disease.

MCI = Mild cognitive impairment − Objective evidence of impairment in one or more cognitive domains, including memory, with preservation of independence in daily life activities

Mild dementia − Substantial progressive impairment affecting multiple cognitive domains, documented by the individual’s report, observer (e.g., partner) report, or change on longitudinal cognitive testing, with clearly evident functional impact on daily life activities

Moderate dementia − Progressive cognitive impairment and extensive functional impact on daily life, with impairment in basic activities

Albert MS, DeKosky ST, Dickson D et al. (2011) The diagnosis of mild cognitive impairment due to Alzheimer's disease: Recommendations from the National Institute on Aging-Alzheimer's Association workgroups on diagnostic guidelines for Alzheimer's disease. Alzheimers Dement 7:270-279. <https://doi.org/10.1016/j.jalz.2011.03.008>

Jack CR, Jr., Bennett DA, Blennow K et al. (2018) NIA-AA Research Framework: Toward a biological definition of Alzheimer's disease. Alzheimers Dement 14:535-562. <https://doi.org/10.1016/j.jalz.2018.02.018>
